# Overview of pediatric and adult lysosomal acid lipase deficiency: expert recommendations from a Gulf cooperation council working group

**DOI:** 10.1186/s13023-025-04158-5

**Published:** 2025-12-05

**Authors:** Moeenaldeen AlSayed, Khalid Al Rasadi, Noura S. AlDhaheri, Abdulrahman Al-Hussaini, Ali Awaji, Amal Al Tenaiji, Khalid Ibrahim Bzeizi, Mohamad Miqdady, Nadia Al Hashmi, Majid Alfadhel

**Affiliations:** 1 Department of Medical Genomics, King Faisal Specialist Hospital and Research Centre, Riyadh, Kingdom of Saudi Arabia; 2 Faculty of Medicine, Alfaisal University, Riyadh, Kingdom of Saudi Arabia; 3 Department of Biochemistry, College of Medicine and Health Sciences, Sultan Qaboos University, Muscat, Oman; 4 Department of Genetics and Genomics, College of Medicine and Health Sciences, United Arab Emirates University, Al Ain, United Arab Emirates; 5 Genetic Division, Pediatrics Department, Tawam Hospital, Al Ain, United Arab Emirates; 6 Children’s Specialized Hospital, King Fahad Medical City, Riyadh, Kingdom of Saudi Arabia; 7 Genetic Center, Prince Mohammed Ben Naser Hospital, Jazan, Kingdom of Saudi Arabia; 8 Sheikh Khalifa Medical City, Abu Dhabi, United Arab Emirates; 9 Department of Liver Transplantation, King Faisal Specialist Hospital and Research Center, Riyadh, Saudi Arabia; 10 Pediatric Gastroenterology, Hepatology, and Nutrition, College of Medicine and Health Sciences, Khalifa University, Abu Dhabi, United Arab Emirates; 11 National Genetic Center, and Department of Pediatrics, Royal Hospital, Muscat, Oman; 12 Genetic and Precision Medicine Department, King Abdullah Specialized Children Hospital, King Abdulaziz Medical City, Ministry of National Guard Health Affairs (MNGHA), Riyadh, Saudi Arabia; 13 Medical Genomic Research Department, King Abdullah International Medical Research Center (KAIMRC), King Saud Bin Abdulaziz University for Health Sciences (KSAU-HS), Riyadh, Kingdom of Saudi Arabia

**Keywords:** Cholesteryl ester storage disease, Dyslipidemia, Hepatomegaly, Infantile lysosomal acid lipase deficiency, Lysosomal acid lipase deficiency, Lysosomal storage disease, Wolman disease

## Abstract

**Background:**

Lysosomal acid lipase deficiency (LAL-D) is an autosomal recessive ultrarare lysosomal storage disease caused by pathogenic/likely pathogenic variants in the *LIPA* gene. The age of onset and progression rate can significantly vary, possibly due to the nature of the underlying variants. The disorder is often misdiagnosed or undiagnosed in the Gulf Cooperation Council (GCC) countries owing to its nonspecific clinical presentation; this necessitates establishing campaigns to increase awareness among healthcare professionals and strategies for identifying and screening high-risk populations. This narrative review is based on an analysis of the available literature, complemented by key discussions among a group of recognized healthcare professionals from the GCC region with expertise in clinical genetics, hepatology, gastroenterology, and lipidology. The outcome of their discussions is a set of practical recommendations and insights aimed at assisting physicians across multiple specialties in the identification and management of individuals affected by this ultrarare genetic disorder.

**Conclusion:**

LAL-D presents significant diagnostic and management challenges, particularly within the GCC region, owing to its rarity, limited awareness, and insufficient utilization of genetic testing. The prevalence and distribution of genetic variations associated with LAL-D remain inadequately explored in this population. The development of standardized regional guidelines is essential to harmonize diagnostic and management practices. Continued research efforts focusing on the genetic landscape of LAL-D in the GCC are imperative to bridge knowledge gaps and enhance clinical outcomes for affected patients.

## Background

Lysosomal acid lipase deficiency (LAL-D; OMIM # 278,000 [1]) is an ultrarare autosomal recessive lysosomal storage disorder caused by pathogenic variants in the *LIPA* gene (OMIM #613497 [1]), which encodes the lysosomal acid lipase (LAL) enzyme [2, 3]. The LAL enzyme is essential for lipid metabolism and catalyzes the hydrolysis of cholesteryl esters (CEs) and triglycerides (TGs) within lysosomes [4]. LAL-D is characterized by the progressive accumulation of lipids in hepatocytes, macrophages, and various other cells, tissues, and organ systems, leading to its clinical manifestations and potentially life-threatening complications [3, 5].

LAL-D manifests in different age groups with varying clinical presentations and is categorized into two subtypes on the basis of the age of symptom onset. Disease severity in LAL-D is largely determined by the level of residual enzyme activity. The early-onset and more severe form, historically called Wolman disease (WD), typically appears during infancy and is characterized by severe intestinal malabsorption, adrenal calcification, hepatomegaly, and early mortality. In contrast, the late-onset form known as cholesteryl ester storage disease (CESD) typically appears in childhood or adulthood and is associated with liver fibrosis, dyslipidemia, and complications such as premature atherosclerosis and liver cirrhosis [3].

The emergence and approval of enzyme replacement therapy (ERT) with Sebelipase alfa marked a significant change in the disease trajectory and transitioned the management focus from nonspecific supportive care to therapy directly addressing enzyme deficiency, the root cause of the condition [6]. It has demonstrated effectiveness in improving survival and development as well as reducing disease-related hepatic and lipid abnormalities [7–9]. Prior to the introduction of ERT, management was limited to supportive care, such as nutritional optimization and lipid-lowering therapies [2], as well as liver transplantation in patients with advanced disease. However, available data on transplantation outcomes are limited, and initial reports suggest no significant alteration in the overall disease course [3, 10].

Currently, LAL-D is underdiagnosed owing to a lack of understanding of the seriousness of disease-related complications [5, 11]. LAL-D presents significant challenges due to the overlap of its signs and symptoms with those of more common conditions. Furthermore, the discrepancy between the clinical and epidemiological data and the genetic prevalence of the disease suggests that LAL-D is particularly underdiagnosed within the Gulf region. Balancing scientific advancements with practical realities is therefore essential to improving patient care, and urgent action is needed to establish clinical guidelines for screening, diagnosing, managing, and long-term monitoring of this life-limiting disease.

## Methodology

A multidisciplinary group of esteemed healthcare specialists in the Gulf Cooperation Council (GCC) region, comprising experts in clinical genetics, hepatology, gastroenterology, and lipidology, convened in an advisory board meeting. Their discussions entailed a comprehensive review of published literature on LAL-D, pertinent guidelines for related conditions, and their collective clinical experiences in evaluating and managing patients with LAL-D. The primary objective was to establish evidence-based best practices for the screening, diagnosis, treatment, and long-term monitoring of LAL-D across all age groups, with particular emphasis on the unique needs of the GCC region.

This article is a narrative review of recent literature, including studies, guidelines, registries, and expert recommendations. This was done via Medical Subject Headings (MeSH) terms or equivalents such as “Lysosomal acid Lipase Deficiency,” “Wolman disease,” & “Cholesteryl Ester Storage Disease” in PubMed Central, PubMed, Medline, and Google Scholar. In addition, the key discussions and insights of the GCC experts and members of the “Lysosomal Acid Lipase Deficiency in the GCC” advisory board meeting, held on the 24^th^ of February 2024, were incorporated as expert opinions on LAL-D.

## Epidemiology

LAL-D is a rare disease with an unknown prevalence [2, 3, 12]. The exon 8 splice junction mutation (E8SJM), the most common *LIPA* variant associated with LAL-D, has been used to estimate a prevalence as high as 1 per 40,000 [3, 13]. However, this estimate is at odds with the relatively low number of reported cases, suggesting significant underdiagnosis of the disease [3, 14]. Globally, the prevalence of LAL-D has been estimated at 1 per 177,452, which is considerably lower than earlier projections [12]. However, data on the epidemiology of LAL-D in the Gulf region remain scarce. Consanguinity, which is prevalent in the region, may contribute to a higher birth prevalence of autosomal recessive diseases such as LAL-D. Therefore, the true prevalence in the Gulf and surrounding areas is likely underreported, and further research is needed to gain a clearer understanding of the epidemiology of LAL-D in these populations.

### Experts’ Insights

The expert panel emphasized the critical need for accurate epidemiological data on the incidence and prevalence of LAL-D to enhance diagnosis and management. However, achieving consistency in demographic and epidemiological data across the GCC remains challenging, with significant intercountry variation. For instance, Saudi Arabia reported a higher prevalence of the infantile form of LAL-D, whereas in Oman, the late-onset form is more readily diagnosed. In contrast, the United Arab Emirates (UAE) has a relatively low but balanced distribution between the two forms. Compared with global prevalence estimates, the burden of LAL-D in the GCC appears lower, most likely reflecting underdiagnosis rather than a true difference in incidence. These discrepancies highlight the lack of a unified and effective diagnostic approach, potentially contributing to delays in or misdiagnosis of LAL-D in the region. The panel noted that greater awareness, unified guidelines, and improved access to diagnostic testing are needed to generate more accurate prevalence data. The true incidence of LAL-D may remain unclear unless pilot or generalized newborn screening for this disease is implemented.

## Inheritance and genetics

The pathogenic variants associated with LAL-D show notable variability, as observed both in global data and expert anecdotal observations from the Gulf. LAL-D arises from pathogenic *LIPA* gene variants (10q23.31), leading to reduced LAL activity [15]. Based on the Genomenon Mastermind database, the *LIPA* gene has ~2500 published variants [16]. The ClinVar database identified 686 germline variants, of which 171 were classified as “pathogenic” (*n* = 91) or “likely pathogenic” (*n* = 88) [17]. The E8SJM (c.894 G > A) mutation is the most common, accounting for 50–60% of all pathogenic LAL alleles [3].

Severe genetic alterations, such as nonsense variants, frameshift mutations, and point mutations resulting in stop codons, are typically found in infants with LAL-D [18], leading to minimal or absent residual enzyme activity [18]. In contrast, variants that allow residual LAL activity are more frequently observed in children and adults, resulting in later-onset phenotypes [18]. These findings highlight the genetic heterogeneity of *LIPA* variants and emphasize the need for local registries. 

### Experts’ insights

The expert panel highlighted that detailed genetic data on LAL-D in the GCC are limited. Based on their clinical experience, they have observed distinct *LIPA* variants across the region. These include c.607 G > A (missense; Oman), c.260 G > T (p.Gly87Val; Saudi Arabia), and c.530C > T (missense; p.Thr177Ile; UAE). While c.260 G > T, c.530C > T have been reported in published literature and ClinVar [19–21], the c.607 G > A variant has now been described in an Omani cohort, where it was found in 70% (14/20) of affected individuals and in 25% (5/20) of carriers [22]. These findings suggest that certain LIPA variants may be more prevalent in Gulf populations; however, further research and global comparison are needed before a conclusion can be drawn. These observations underscore the urgent need for regional genetic registries and further research to characterize these variants, their frequency, and their influence on the clinical onset and presentation of LAL-D in the GCC.

## Clinical manifestations

The disease severity and rate of progression are influenced by the underlying variants, residual LAL enzyme activity, and age of onset [10]. However, no genotype‒phenotype correlation has been clearly identified, and a compelling hypothesis that requires further validation remains.

Drawing from experience with patients in the GCC region, the expert panel noted that LAL-D manifests in diverse ways, both clinically and biochemically. These include hepatosplenomegaly, chronic diarrhea, failure to thrive (FTT), cachexia, pancytopenia, high serum ferritin, elevated low-density lipoprotein-cholesterol (LDL-C) and triglycerides (TG) levels, and osteopenia due to malnutrition. These manifestations are consistent with those described in international cohorts, as shown in analyses of baseline data from children and adults with LAL-D enrolled in global registries, including patients from Saudi Arabia [23].

LAL-D is traditionally categorized into two phenotypes: the severe infantile-onset WD and the milder late-onset CESD. Their distinguishing features are summarized in Table 1. No distinct phenotypic differences between Gulf patients and those from other regions have been established, though the limited regional data highlight the importance of further study.

**Table 1:** Clinical presentation of LAL-D phenotypes [2, 3, 14, 15, 24–29]

	Infantile-onset LAL-D (WD)	Late-onset LAL-D (CESD)
Onset	First days to months of life	Childhood to adulthood
Residual LAL activity	< 1%	1–12%
Main organs affected	Liver, spleen, adrenal glands, bone marrow, lymph nodes, intestinal villi	Primarily liver; also gallbladder, adrenal glands, vasculature
Key clinical features	GI symptoms (vomiting, diarrhea, abdominal distension), massive hepatosplenomegaly, FTT, adrenal calcification	Hyperlipidemia, hepatomegaly, elevated liver enzymes, gallbladder disease, cholestasis. GI symptoms: diarrhea/steatorrhea, vomiting, abdominal pain, and malabsorption in some children
Course	Rapid progression, multi-organ involvement	slower progression; often detected incidentally
Outcome	Early death in infancy without treatment	Premature death due to liver failure with or without accelerated atherosclerosis

CESD, cholesteryl ester storage disease; FTT, failure to thrive; GI, gastrointestinal; LAL, lysosomal acid lipase; LAL-D, lysosomal acid lipase deficiency; WD, Wolman disease

### Experts’ insights

The expert panel acknowledged that in the absence of early identification and treatment, patients affected by the severe infantile form of LAL-D in the GCC region typically do not survive beyond their first year of life. The panel highlighted that adrenal calcification is a common characteristic feature of infantile-onset LAL-D. As reported in several studies, radiological images may show calcification of the adrenal glands in approximately 50–70% of affected infants [2, 3, 24]. Nevertheless, this finding alone is clinically insufficient in ruling out the condition since it is not present in all patients, emphasizing the need for additional clinical and biochemical evaluation in the diagnostic process. Moreover, secondary hemophagocytic lymphohistiocytosis (HLH) is an important manifestation that may be overlooked until it is more severe and can be a misdiagnosis for infantile LAL-D.

The expert panel emphasized that liver injury markers and dyslipidemia represent the predominant clinical features of late-onset disease. Furthermore, they supported their remarks with existing evidence from the literature that distinguishes age-specific manifestations and highlighted that both forms of LAL-D show elevated lipid markers (CEs and TG) and reduced LAL enzyme activity. However, the extent of lipid elevation and enzyme deficiency varies, with lower LAL enzyme activity identified as a key driver of symptom exacerbation [2, 3, 5, 30, 31].

## Disease prognosis

LAL-D is a rapidly progressive disorder in infants, often resulting in death by six months of age if left untreated [2, 15, 32]. The natural history data indicate a median age at death of 3.7 months, with no survivors among 35 infants beyond 48 months [32]. The primary causes of mortality in this population are malnutrition and liver disease [15]. A case series involving seven infants with LAL-D in Egypt identified complicated chest infections and chronic diarrhea as the causes of death [33].

In certain cases, liver transplantation becomes necessary, particularly when children present with early liver failure [3]. An observational study involving 32 children and adults revealed that the median duration from the first clinical manifestation of LAL-D to the onset of hepatic fibrosis, cirrhosis, or liver transplantation was 3.1 years [34]. Approximately 65% of patients with LAL-D develop fibrosis or cirrhosis, with 13% requiring liver transplantation before the age of 44 [35]. Furthermore, LAL-D is associated with an increased risk of tumors, such as hepatocellular carcinoma, and cardiovascular issues, including coronary artery disease and stroke [4].

The expert panel highlighted the limited number of identified LAL-D patients in the GCC region, which is closely linked to the poor prognosis of LAL-D patients. This is attributed to the broad range of clinical manifestations that often lead to misdiagnosis, as well as the lack of awareness of the disease’s characteristics in the region, further contributing to delayed diagnosis and a greater risk of fatal complications.

## Diagnosis of LAL-D

LAL-D is frequently misdiagnosed due to several factors, including its rarity, limited awareness, and overlap of its nonspecific clinical features with those of other more common conditions. The manifestations of LAL-D, such as dyslipidemia, hepatomegaly, elevated liver enzymes, and GI symptoms, often mimic those observed in cardiovascular, hepatic, and metabolic disorders [5].

### Diagnostic challenges in the GCC region

Experts have discussed several diagnostic challenges in the GCC region, including overlooking family history by nongeneticist healthcare professionals who lack the genetic expertise to differentiate LAL-D from other disorders. Additionally, the late-onset form of LAL-D is less defined and more challenging to diagnose than the severe infantile form. Even in cases of infantile LAL-D, where symptoms may be apparent, the diagnosis remains complex, necessitating careful differential diagnosis.

They also stress the need to promptly refer suspected LAL-D cases, particularly infants presenting with FTT, to specialized healthcare providers. They discussed the potential for targeted awareness initiatives and newborn screening programs for LAL-D, particularly in regions with a higher prevalence of the condition. However, they debated the balance between the potential benefits of such programs and the stigma they might impose on affected populations. A unique regional factor contributing to the prevalence of LAL-D and other inborn errors in metabolism is the tradition of consanguineous marriage, particularly within families and tribal communities [36]. Despite this, many of these conditions remain underdiagnosed owing to the lack of biochemical and molecular genetic diagnostic facilities and the absence of a comprehensive disease registry [36]. To address these challenges, the experts pointed out several strategic initiatives, such as increased awareness of LAL-D symptomatology, improved access to specialized biochemical and genetic testing, and enhanced referral pathways to tertiary rare disease centers. Moreover, they advocated for efforts to address the systemic gaps in the diagnostic infrastructure, including the establishment of a regional disease registry to enhance early and accurate diagnosis of LAL-D in the GCC region.

### Differential diagnosis

The clinical manifestations of LAL-D often resemble those of more prevalent diseases, including familial hypercholesterolemia (FH), HLH, metabolic dysfunction-associated steatotic liver disease (MASLD, the updated term for nonalcoholic fatty liver disease, NAFLD) [37, 38], metabolic dysfunction-associated steatohepatitis (MASH, the updated term for nonalcoholic steatohepatitis, NASH) [37, 38], Niemann‒Pick disease, Gaucher disease, and cryptogenic cirrhosis [2, 3, 5, 39–42].

The expert panel emphasized that almost all pediatric and adult patients with LAL-D present with high LDL-C and/or low HDL-C levels. These lipid abnormalities should serve as the primary indicators for differential diagnosis and guide the diagnostic process to confirm LAL-D [5]. Given the frequent overlap in clinical features, misdiagnosis is common. The most relevant differentials in the Gulf region include FH, liver diseases, and HLH. Table 2 summarizes the main overlapping features, distinguishing characteristics, and expert insights, providing a concise framework for clinicians.

**Table 2:** Key differential diagnoses of LAL-D and expert insights from the GCC [3, 5, 14, 40, 41, 43–46]

Feature	Familial Hypercholesterolemia	Liver Diseases	Hemophagocytic Lymphohistiocytosis
Overlap with LAL-D	Dyslipidemia (high LDL-C, overlapping HDL-C levels)	Hepatomegaly, elevated transaminases	FTT, hepatosplenomegaly, adrenal calcification
Distinguishing features	Autosomal dominant inheritance, strong family history, no liver enzyme elevation	Often obesity/metabolic syndrome, viral/autoimmune markers	Immune dysregulation syndrome, less pronounced hepatosplenomegaly
Diagnostic workup	Lipid profile, liver function testing, family history, genetic testing, LAL enzyme activity	Viral hepatitis serology, autoimmune workup, enzyme/genetic testing for LAL-D	Bone marrow exam, immune studies, exclude metabolic disorders
Expert insights (GCC)	Frequently misdiagnosed in GCC due to overlapping dyslipidemia; elevated transaminases point to LAL-D; FH usually has clear family history; assess patient with overlapping symptoms who tested FH negative for LAL-D; Molecular diagnostics are essential for identifying genetic causes of dyslipidemia and confirming LAL-D.	In GCC, non-obese patients with unexplained liver abnormalities should raise suspicion of LAL-D; This is a high-risk group that should be evaluated for LAL enzyme activity or genetic testing.	Infants with HLH-like symptoms but pronounced hepatosplenomegaly should be evaluated for LAL-D

FH, Familial Hypercholesterolemia; FTT, Failure to Thrive; GCC, Gulf Cooperation Council; HDL-C, High-Density; HLH, Hemophagocytic Lymphohistiocytosis; HSCT, Hematopoietic Stem Cell Transplantation; Lipoprotein-Cholesterol; LAL, Lysosomal Acid Lipase; LAL-D, Lysosomal Acid Lipase Deficiency; LDL-C, Low-Density Lipoprotein-Cholesterol

### Screening recommendations: keys to the early identification of LAL-D

**Fig. 1:**
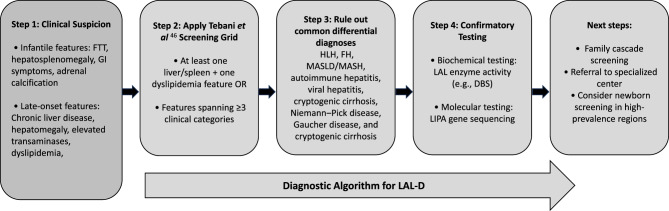
Proposed diagnostic algorithm for LAL-D in the GCC region

In 2021, Tebani et al. [47] developed a screening criteria grid for LAL-D on the basis of clinical and biological data, with the aim of improving diagnostic accuracy in at-risk populations [47]. To provide a concise and practical overview, we incorporated the key elements of this grid into a diagnostic algorithm, alongside expert panel recommendations (Fig. 1).

### Experts’ recommendations for LAL-D screening

The expert panel highlighted that the collaboration of a multidisciplinary team, including pediatricians, primary healthcare providers, lipidologists, cardiologists, gastroenterologists, hepatologists, and geneticists, is essential for diagnosing and caring for patients with LAL-D. Additionally, mining electronic medical records and strategies to flag laboratory results suggestive of LAL-D would increase awareness and link providers to regional specialist centers for the management of patients with LAL-D.

To improve LAL-D diagnosis rates in the GCC region, the expert panel strongly recommends the following: (i) establishing newborn screening programs for early detection of LAL-D in addition to fostering national screening programs—involving biochemical or molecular testing—targeting the high-risk patient population; (ii) screening for LAL-D should be conducted whenever clinically suspected following the screening criteria recommended by Tebani et al. [47], in addition to cascade screening for the family members of patients with LAL-D; (iii) providing premarital screening in high-prevalence areas in the Middle East and the GCC; (iv) implementing educational programs to increase awareness among all healthcare providers; and (v) generating additional evidence to highlight the high-risk group.

The expert panel emphasized that translating these recommendations into practice within the GCC must be considered in light of the diagnostic challenges described in section “Diagnostic challenges in the GCC region:”. These include limited awareness among healthcare providers, restricted and unequal access to biochemical and molecular diagnostic facilities, absence of national registries, variability in healthcare infrastructure across countries, and the influence of cultural factors such as consanguinity. Addressing these barriers is critical to ensuring the feasibility and impact of screening initiatives.

### Diagnostic tests

Detecting deficient LAL activity and *LIPA* gene variants is the primary diagnostic method for LAL-D [3]. Moreover, biopsy and radiological findings can raise suspicion of LAL-D [5].

#### Measurement of LAL activity

A widely adopted method involves a dried blood spot (DBS) assay that uses Lalistat 2, a highly specific inhibitor of LAL. The enzyme activity is measured by comparing total lipase activity with lipase activity in the presence of Lalistat 2. This technique is reliable, requires only a small sample size, and allows transport at ambient temperature, with good sample stability [48]. It enables rapid identification of individuals with reduced enzyme activity, thereby supporting screening initiatives in both high-risk populations and potential newborn programs [48].

The expert panel recommends using DBS testing to rapidly and reliably assess LAL enzyme activity for screening and diagnosing LAL-D. However, no laboratories in the Middle East and North Africa or the GCC region currently perform this test. Therefore, the panel agrees that genetic testing for pathogenic *LIPA* variants should be initiated concurrently rather than sequentially, given that (i) LAL enzyme activity testing may yield false-negative results, (ii) genetic testing is more widely accessible in the region, and (iii) rapid genomic testing capabilities are now available in several GCC diagnostic laboratories. This approach is particularly important for early diagnosis of the infantile-onset form, allowing prompt initiation of ERT and improved clinical outcomes.

#### Genetic testing

Sequencing of the *LIPA* gene can help identify variants in individuals with suspected LAL-D [11]. The expert panel recommended *LIPA* gene sequencing for LAL-D confirmation, particularly in patients with diseases with overlapping clinical manifestations. Broader next-generation sequencing–based metabolic panels – covering genes associated with inborn errors of metabolism – may also be considered in the differential diagnosis to rule out other metabolic conditions that mimic LAL-D. The experts also emphasized the necessity for the availability of LAL enzyme activity measurement and genetic testing methodologies in the GCC region to enable swift and efficient detection of the condition.

#### Liver biopsy

Liver biopsy is widely acknowledged as the most reliable approach for assessing liver irregularities. Nevertheless, its implementation is associated with potential risks, substantial costs, and the likelihood of sampling inaccuracies. Contemporary guidelines advocate the initial utilization of noninvasive methodologies, reserving the biopsy procedure for instances where alternative methods yield inconclusive results [49, 50]. Histological indicators are essential to validate the diagnosis of LAL-D, including hypertrophic Kupffer cells, ceroid-laden, and portal macrophages exhibiting foamy, tan-colored cytoplasm [5, 51]. Identifying luminal and membrane lysosomal markers surrounding lipid vacuoles and the presence of CE crystals in samples serve as indicative evidence supporting the diagnosis of LAL-D [39].

The expert panel highlighted the potential of studying high-risk populations within databases to help identify patients with specific laboratory results or certain biopsy findings and allow targeted testing for LAL-D. They emphasized that liver biopsy, while essential in selected cases, should ideally be complemented by noninvasive tests and monitoring in order to better characterize disease progression and guide management in LAL-D.

#### Radiological techniques

Caution is warranted when interpreting fibrosis staging using noninvasive alternatives, such as transient elastography, shear-wave elastography, or magnetic resonance elastography. Although these modalities are widely used in metabolic liver diseases, their accuracy in LAL-D remains uncertain, as fibrosis may be underestimated owing to massive hepatic lipid infiltration [52]. A 3T magnetic resonance imaging scanner for hepatic magnetic resonance spectroscopy has recently been identified as an effective noninvasive technique for detecting and measuring the specific hepatic lipid patterns associated with LAL-D [53]. This method may present a more effective alternative to frequent biopsy sampling for clinical assessment [5]. The expert panel noted that ultrasound diagnosis of a fatty liver can be subjective, making it an unreliable tool for detecting liver alterations in patients with LAL-D. In patients with WD, the suspicion of LAL-D can be supported by an abdominal computed tomography scan showing enlarged adrenal glands with calcifications.

## Treatment and management approaches

There are currently no widely accepted treatment protocols for LAL-D. Continuous evaluation of the clinical characteristics of LAL-D and their associated laboratory findings could assist in optimizing clinical care, incorporating recent advancements in therapy, and improving outcomes [51]. No disease-specific treatments were previously available for LAL-D; historical approaches focused on supportive therapies only [5]. However, the expert panel highlighted that these approaches do not address the underlying pathophysiology of LAL-D and do not prevent the progression of the disease. ERT, sebelipase alfa, is currently the standard treatment for LAL-D, providing proven evidence of reducing multiple disease-related hepatic and lipid abnormalities by achieving near-physiological enzyme levels, preventing the accumulation of lipids, and restoring normal organ function [5]. A brief description of the current treatment approaches for LAL-D is depicted in Table 3 [5, 54–58].

**Table 3:** Conventional treatments of LAL-D and ERT

Treatment	Mechanism of Action	Effects on LAL-D	Limitations
Statins [54]	HMG-CoA reductase inhibition	Reduces LDL-C and TG	No effect on lysosomal lipid accumulation; does not improve liver disease
HSCT [55]	Supporting improvement of the acid lipase activity by allogeneic bone marrow transplantation	Can provide benefits when considered in suboptimal responders to ERT	High risk, limited by donor availability, not sustainable, and requires continuing ERT
PCSK9 inhibitors [56, 57]	Inhibits PCSK9, increases LDLR activity	Reduces LDL-C	No impact on hepatic lipid storage or liver function
ERT (Sebelipase alfa) [5, 58]	Replaces deficient LAL	Reduces LDL-C and TG; clears lysosomal lipid accumulation; improves liver function	Requires lifelong administration and cost.

ERT, Enzyme Replacement Therapy; HMG-CoA, Hydroxymethylglutaryl Coenzyme A; HSCT, Hematopoietic Stem Cell Transplantation; LAL, Lysosomal Acid Lipase; LAL-D, Lysosomal Acid Lipase Deficiency; LDL-C, Low-Density Lipoprotein-Cholesterol; LDLR, Low-Density Lipoprotein Receptor; PCSK9 inhibitors, Proprotein convertase subtilisin/kexin type 9 inhibitors; TG, Triglycerides

### Dietary management and supportive care

Proper nutrition is essential for patients with LAL-D. In infancy, many patients require temporary parenteral nutrition because of significant malabsorption. A nutrition team should guide dietary support, with emphasis on low-cholesterol and low-triglyceride diet [15]. Close monitoring is needed to avoid essential fatty acid deficiency, especially in WD, where supplementation with medium-chain triglyceride oil may be necessary. The medical literature highlights that while dietary interventions and lipid-lowering therapies reduce CE accumulation, these approaches show limited success when used alone, as they do not target the root cause of the disease [51, 59].

### Hematopoietic stem cell transplantation and liver transplantation

Evidence for the use of HSCT and/or liver transplantation in LAL-D remains limited and conflicting. While liver transplantation can save the lives of patients with liver failure, it does not address the underlying enzyme deficiency or prevent systemic disease progression [3, 10, 60, 61]. Similarly, HSCT has shown limited success because of the disease’s multisystem nature, high toxicity, and challenges with sustained engraftment in target tissues [62, 63], though some benefit has been reported when combined with pretransplant ERT [64]. The expert panel recommends considering HSCT in infantile LAL-D in cases of suboptimal response to ERT, especially in patients with severe GI symptoms and growth failure [65].

### Lipid-lowering medications

Treating the underlying LAL deficiency is crucial for restoring cholesterol homeostasis in these patients. Additionally, lipid-lowering medications have no or little effect on cardiovascular disease risk, as Sebelipase alfa can lead to further reductions in lipid levels [66]. Statins reduce cholesterol synthesis, decreasing the production of Apolipoprotein B and increasing the expression of low-density lipoprotein receptors (LDLRs). This leads to increased uptake of plasma LDL-C and accelerates the accumulation of CEs in lysosomes, potentially affecting liver function [5]. The expert panel recognized that LAL-D patients on statins may experience continued elevation of serum transaminases and progression of liver fibrosis to cirrhosis [3, 67].

PCSK9 inhibitors have been considered adjuncts to lipid-lowering therapy, particularly in patients with severe dyslipidemia who do not respond adequately to statins. However, for LAL-D, experts reported that while PCSK9 inhibitors reduce LDL-C levels, they do not address lysosomal lipid accumulation in the liver.

### Enzyme replacement therapy (Sebelipase Alfa)

Sebelipase Alfa, a recombinant human lysosomal acid lipase, represents a major advance in the management of LAL-D and is currently the only approved therapy worldwide [68, 69]. Clinical trials and real-world studies consistently demonstrated that sebelipase alfa is well tolerated, substantially improves survival and clinical outcomes across age groups [7–9, 32, 58, 66, 70, 70–73].

In infants with the rapidly progressive form, pooled data from the CL03/VITAL and CL08 studies showed a 79% survival rate at 12 months and 68% at 5 years [9], accompanied by rapid and sustained reductions in liver injury markers and improvements in growth parameters [7, 9]. In the childhood and adult forms of LAL-D, treatment with sebelipase alfa was associated with short-term and long-term improvements in aspartate aminotransferase (AST) and alanine aminotransferase (ALT) levels. It also improved atherogenic biomarkers regardless of lipid lowering medications use [66], and liver disease in various patients [8, 72]. After 20 weeks, the ARISE study revealed improvements in liver fat, steatosis, and volume compared to placebo [58].

Sebelipase alfa is generally well-tolerated; the most commonly reported adverse events include mild to moderate infusion-related reactions, such as headache, pyrexia, and nausea, which are typically manageable [7, 9, 58].

Experts emphasized that timely access to sebelipase alfa can significantly reduce disease-related mortality, particularly in infants with WD, and that it should be considered the standard of care for LAL-D where available. However, access in the GCC region remains limited due to cost and availability. This has led to supportive measures continuing to play a key role.

### Interdisciplinary care plan

A multidisciplinary approach is essential for managing the complex manifestations of LAL-D [51]. The core members of the care team should include metabolic disease specialists, hepatologists, gastroenterologists, cardiologists, lipidologists, and nutritionists [51], each addressing different aspects of the disease [15, 34]. A single coordinating physician, typically a metabolic disease specialist, is recommended to oversee treatment and ensure effective communication within the team [51]. Regular team consultations are vital for evaluating the effectiveness of therapies and adjusting management strategies according to the patient’s condition [32]. Given the progressive nature of LAL-D, a structured transition plan from pediatric to adult care is needed to ensure continuity [2, 51]. Psychological support should also be available for patients and families [51]. The expert panel highlighted that nutritionists play a crucial role in managing the metabolic aspects of LAL-D. Early and ongoing nutritional interventions can help improve long-term outcomes, especially in patients with hepatic or cardiovascular involvement.

### Experts’ Recommendations for LAL-D Treatment

**Fig. 2:**
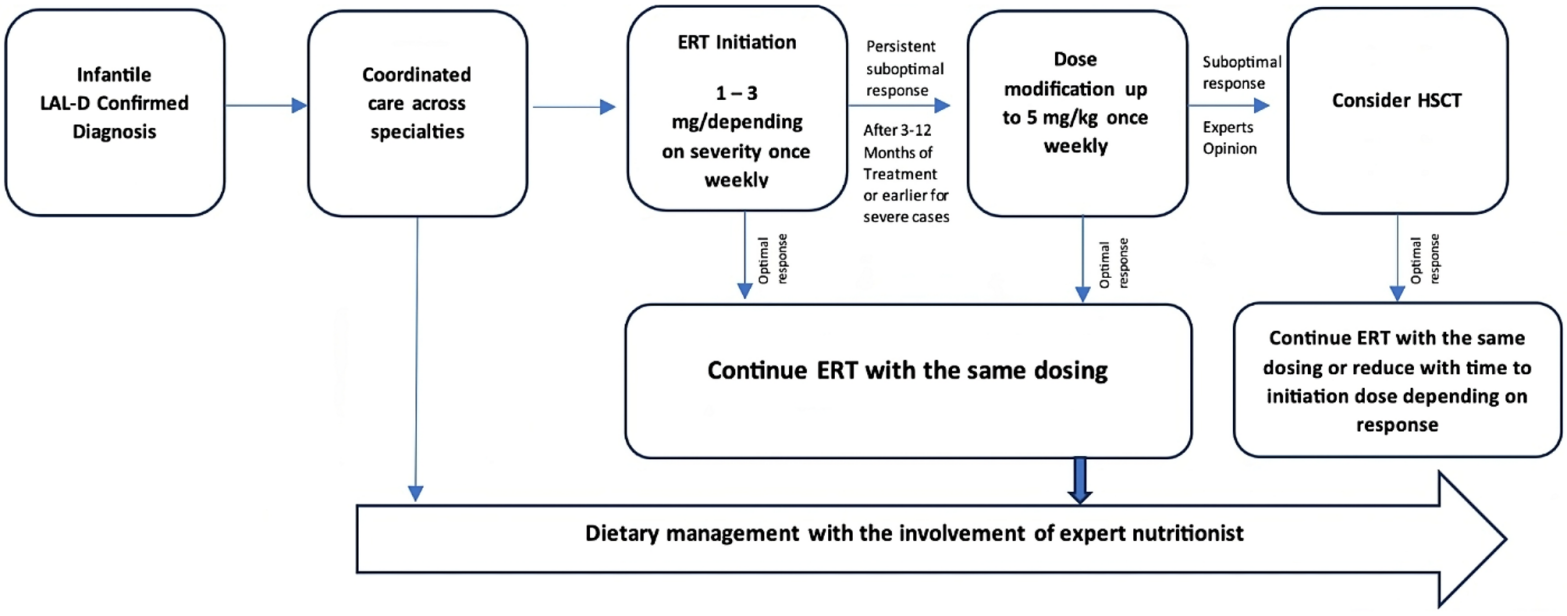
Experts’ recommendations for infantile LAL-D management

For the infantile severe form of LAL-D, the experts outlined a recommended treatment pathway, as summarized in Fig. 2.

For infants under 6 months of age with rapidly progressive LAL-D, the recommended starting dose is 1 mg/kg or 3 mg/kg administered weekly via intravenous infusion, depending on clinical status. A higher initial dose of 3 mg/kg is advised in cases of severe disease or rapid progression. Dose escalation to 3 mg/kg should be considered in the context of a suboptimal clinical response, which may be indicated by poor growth, worsening biochemical markers (e.g., elevated liver transaminases, ferritin, C-reactive protein), persistent or worsening organomegaly, increased frequency of infections, or worsening symptoms [74].

Particularly, within the first three months of treatment, dose escalation is recommended in cases of suboptimal response, defined by at least two criteria, including failure to achieve 5 g/kg/day and either a WHO z score for weight-for-length/height < −2 or length-for-age < −2; albumin < 35 g/L; ALT > 2× the upper limit of normal; or the need for ongoing blood/platelet transfusions [8]. After three months, further dose escalation should be considered for persistent suboptimal responses and unresolved clinical manifestations [7]. However, on the basis of expert experience, dose escalation may be warranted earlier in severe cases.

Generally, for LAL-D management, expert insights can be summarized in the following points: (i) coordinated care across specialties is important to address the wide range of clinical manifestations of LAL-D and to facilitate a smooth transition from pediatric to adult care; (ii) Sebelipase Alfa is strongly recommended for long-term ERT in patients with LAL-D across all age groups; (iii) statins and PCSK9 inhibitors do not address the root cause of LAL-D; they merely partially control lipid abnormalities, offer some protection against atherosclerosis and vascular diseases, and minimally reduce hepatomegaly, all while accelerating liver disease progression; (iv) dietary management with the involvement of an expert nutrition team is essential for patients with LAL-D; and (v) transplantation is considered if there is end-stage organ damage, such as liver failure, or if there are complications that ERT cannot address. HSCT can be added when there is a suboptimal response to ERT.

From a regional perspective, experts noted several challenges, including limited referral pathways to specialized centers, lack of access and funding for long-term ERT, and gaps in structured monitoring and follow-up programs. However, they mentioned opportunities that include the presence of specialized rare and metabolic disease centers to coordinate care and improve outcomes. A further challenge in the GCC region is the absence of formal patient advocacy groups for LAL-D, which contrasts with other rare diseases where such organizations play a key role in raising awareness, supporting families, and facilitating access to care. The experts’ vision is to not only improve survival outcomes but also enhance long-term quality of life through early diagnosis, coordinated care, and patient support.

## Disease monitoring

Conducting annual evaluations for patients with LAL-D is crucial for monitoring disease progression and evaluating the safety and effectiveness of therapeutic interventions [3, 51]. The recommended assessment parameters include laboratory investigations to monitor dyslipidemia and liver disease. On the basis of clinical judgment, imaging techniques should be employed to monitor liver fibrosis, portal hypertension, and the risk of variceal bleeding. A follow-up liver biopsy to evaluate disease progression or improvement may be recommended on the basis of the treating clinician’s judgment. Additionally, it is advisable to perform annual cardiovascular and neurovascular evaluations to assess cardiovascular complications and the risk of stroke. Renal and hematologic assessments are also recommended to determine kidney disease risk and follow up on splenomegaly [3, 51].

## Conclusion

LAL-D is a rare disorder with variable onset and phenotypic severity, often leading to significant underdiagnosis owing to its nonspecific clinical presentation. Infantile-onset LAL-D is fatal within the first year of life, whereas childhood- and adult-onset forms are associated with reduced life expectancy due to severe liver disease, early liver transplantation, and increased risk of early-onset cardiovascular disease from dyslipidemia and accelerated atherosclerosis.

Timely diagnosis is critical, with proposed criteria including LAL enzyme activity measurement and genetic testing. Current management emphasizes lipid control and addressing liver complications, but the limited efficacy of lipid-lowering medications highlights the need for targeted therapies such as Sebelipase alfa. Raising awareness of LAL-D among healthcare providers is essential to ensure its consideration in differential diagnoses. Broad enzyme activity screening and genetic testing in pediatric and adult patients with liver disease or dyslipidemia are recommended to facilitate early diagnosis and intervention.

To standardize care, the development of national guidelines for screening, diagnosis, management, and monitoring of LAL-D is advised. Regular updates to these guidelines will be necessary as new research and real-world clinical insights become available.

## Data Availability

Not applicable.

## Abbreviations

ALT: Alanine Aminotransferase
AST: Aspartate Aminotransferase
CE: Cholesteryl Ester
CESD: Cholesteryl Ester Storage Disease
ERT: Enzyme Replacement Therapy
E8SJM: Exon 8 splice junction mutation
FH: Familial Hypercholesterolemia
FTT: Failure to Thrive
GCC: Gulf Cooperation Council
GI: Gastrointestinal
HDL-C: High-Density Lipoprotein-Cholesterol
HLH: Hemophagocytic Lymphohistiocytosis
HMG-CoA: Hydroxymethylglutaryl Coenzyme A
HSCT: Hematopoietic Stem Cell Transplantation
LAL: Lysosomal Acid Lipase
LAL-D: Lysosomal Acid Lipase Deficiency
LDL-C: Low-Density Lipoprotein-Cholesterol
LDLR: Low-Density Lipoprotein Receptor
LIPA: Lipase A, Lysosomal Acid Type
MASH: Metabolic dysfunction-associated steatohepatitis
MASLD: Metabolic dysfunction-Associated Steatotic Liver Disease
NAFLD: Non-Alcoholic Fatty Liver Disease
NASH: Non-Alcoholic Steatohepatitis
TG: Triglycerides
WD: Wolman Disease
